# Impact of High Maximum Dose Constraints Within the Gross Tumor Volume on the Quality of Stereotactic Radiosurgery Plans Using Volumetric-Modulated Arcs for Brain Metastases

**DOI:** 10.7759/cureus.88548

**Published:** 2025-07-22

**Authors:** Kazuhiro Ohtakara, Kojiro Suzuki

**Affiliations:** 1 Department of Radiation Oncology, Kainan Hospital Aichi Prefectural Welfare Federation of Agricultural Cooperatives, Yatomi, JPN; 2 Department of Radiology, Aichi Medical University, Nagakute, JPN

**Keywords:** brain metastases, dose conformity, dose constraint, dose gradient, dose inhomogeneity, internal dose escalation, internal high dose, stereotactic radiosurgery, treatment planning time, volumetric-modulated arc therapy

## Abstract

Purpose

This planning study aimed to clarify the significance of maximum dose constraints within a gross tumor volume (GTV), exceeding 1.5 times the prescription dose, in stereotactic radiosurgery (SRS) using volumetric-modulated arcs (VMA) with the Monaco® planning system (Elekta AB, Stockholm, Sweden) for single brain metastases (BMs).

Materials and methods

Thirty five lesions were included with the GTV ranging from 0.33 cc to 48.09 cc (median 7.34 cc). Four VMA plans were developed for each GTV using a multileaf collimator (MLC) Agility^®^ (Elekta AB): one without any maximum dose constraint (None group) and three with increasing constraints (55%, 60%, and 65% groups). In the 55%, 60%, and 65% groups, maximum dose constraints of ≤181.82%, ≤166.67%, and ≤153.85% of the prescription dose were assigned to achieve the GTV coverage by ≥55%, ≥60%, and ≥65% isodose surfaces (IDSs), respectively. The arc and MLC angle configurations and optimization method are unified, except for the maximum dose constraint. The GTV coverage by the prescription dose was rescaled to the *D*_V-0.01 cc_: the minimum dose to a GTV minus 0.01 cc (*D*_>95%_) for GTV >0.20 cc or *D*_95%_ for GTV ≤0.20 cc.

Results

The total planning time was significantly shorter in the None group. This group showed the most statistically significant superiority in GTV dose conformity, the appropriateness of the dose attenuation margin, and the steepness and concentric lamellarity of dose gradients 2 mm outside and 2-4 mm inside the GTV boundary. The None group also showed favorable trends in the steepness of dose fall-off from the prescription dose to 50%. The 55% and 60% groups showed a favorable trend in smaller prescription isodose volume spillage; however, no significance was observed in other evaluation metrics.

Conclusions

Maximum dose constraints within the GTV, at 1.5-1.8 times the prescription dose, likely impaired GTV dose conformity, the appropriateness of the dose attenuation margin, and the steepness and concentric lamellarity of dose gradients outside and inside the GTV boundary. Furthermore, they deteriorated the efficiency of VMA optimization in maximizing dose conformity and gradients. Therefore, dose constraints to ensure ≥55-65% IDS coverage are not recommended, and alternative methods should be considered to adjust excessively steep dose attenuation margins in small GTVs.

## Introduction

Stereotactic radiosurgery (SRS) is becoming an increasingly important treatment option for brain metastases (BMs), thanks to its minimal invasiveness and high therapeutic effect [1-3]. The appropriate implementation of SRS using a general-purpose linear accelerator (linac) allows for seamless coordination with systemic therapy and rapid delivery of SRS at nearby facilities [4]. There remain substantial opportunities to improve local control and safety, especially for lesions ≥2 cm [5-7]. The two main reasons for local failures are the compromise of lowering the prescription dose for larger lesions and the insistence on ≤5 fractions, with insufficient consideration of the lower brain tolerance dose than previously recognized [8-10]. Adopting ≥5 dose fractions to maintain a consistent biologically effective dose (BED) to the gross tumor volume (GTV) boundary may improve outcomes in treating large BMs [5,11,12]. Furthermore, it is important to design and create an appropriate dose distribution to maximize therapeutic efficacy and safety. The distribution factors include excellent dose conformity, an appropriate dose attenuation margin, and the steepness and concentric lamellarity of dose gradients outside and inside the GTV boundary [13].

Volumetric-modulated arcs (VMA) are an essential technique for maximizing the efficacy and safety of SRS using a 5 mm leaf-width multileaf collimator (MLC), and for expanding the range of application compared with dynamic conformal arcs (DCA) [11-14]. Even when limited to VMA, however, inter-institutional differences in target volume definition, dose distribution, and dose prescription method remain significant [15,16]. In particular, perceptions of optimal target dose heterogeneity vary considerably [15-17]. The prescription dose for VMA or DCA is commonly assigned to a 70-80% isodose surface (IDS), relative to the maximum or central dose, covering the planning target volume (PTV) with a ≥1 mm isotropic margin [15-20]. VMA optimization usually imposes a maximum dose constraint within the PTV [18-23].

At our institution, a simple and efficient VMA optimization method has been investigated to develop the optimal dose distribution for SRS that is physically achievable using the Agility® MLC (Elekta AB, Stockholm, Sweden) [14,24]. Compared with 80-90% IDS coverage, an extremely inhomogeneous GTV dose without maximum dose constraints was shown to be optimal in terms of superior dose conformity and the steepness of the gradient outside the GTV [24]. In some small lesions, however, too steep a dose falloff outside the GTV boundary may result in insufficient control of microscopic brain invasion [25].

This planning study therefore aimed to clarify the significance of maximum dose constraints within a GTV, exceeding 1.5 times the prescription dose, in VMA-based SRS using the Agility® MLC (Elekta AB) and the Monaco® planning system (Elekta AB). Specifically, we investigated the effects of maximum dose constraints, such as covering the GTV with a ≥55-65% IDS, on VMA-based SRS planning.

## Materials and methods

This study was approved by the Clinical Research Review Board of Kainan Hospital Aichi Prefectural Welfare Federation of Agricultural Cooperatives (approval number: 20240830-01).

Thirty-five lesions were extracted from previous cases of brain metastasis (BM) treatment to include a variety of sizes, shapes, and locations, and each was treated as a single lesion. A significant number of the 35 lesions had also been used in previous studies [13]. Each GTV was defined based on the T2/postcontrast T1 matching, as described previously [13]. The GTV ranged from 0.33 cc to 48.09 cc (median value: 7.34 cc; IQR: 2.01, 21.54 cc).

The treatment platform consisted of a flattening filter-free (FFF) mode of a 6-MV X-ray beam provided by a linac Infinity® (Elekta AB, Stockholm, Sweden), a 160-leaf, 5-mm leaf-width MLC Agility® (Elekta AB), a planning system Monaco® Version 5.51.10 (Elekta AB), and a planning support system MIM Maestro® Version 7.1.3 (MIM Software Inc., Cleveland, Ohio, United States) [13,14]. The irradiation isocenter was set at each GTV center. The following uniform arc configurations and collimator angles were applied: one coplanar arc with 360º rotation and a collimator angle of 0º, and two non-coplanar arcs with 180º rotation each, with collimator angles of 45º and 90º to divide the cranial hemisphere into thirds [26,27]. The increment (Inc) parameters of each arc were all set at 20º [13].

Four different SRS plans were developed for each GTV: one without any dose constraint within the GTV and three with different dose constraints within the GTV, as detailed in Table 1.

**Table 1:** Comparison of the four groups with or without maximum dose constraints within the gross tumor volume (GTV). *Prescribed isodose surface (%) relative to the maximum dose. **Maximum dose (%) relative to the prescribed isodose surface.

Group	None	55%	60%	65%
Intended lower limit of % isodose coverage*	No limitation	≥55%	≥60%	≥65%
Intended upper limit of maximum dose**	No limitation	≤181.82%	≤166.67%	≤153.85%
Example of maximum dose constraint (e.g., prescription dose = 43.000 Gy)	None	78.182 Gy	71.667 Gy	66.154 Gy

The optimization method of VMA, established based on previous studies and used in this study, is shown in Table 2 [13].

**Table 2:** Details of the unified optimization settings using the Monaco® planning system. *The minimum volume is set according to the coverage value of *D*_V-0.01 cc_, minimum dose to a target volume (TV) minus 0.01 cc (*D*_>95%_ for TV >0.20 cc, *D*_95%_ for TV ≤0.20 cc), for each GTV. **The Quadratic Overdose cost function is applied to each GTV only in the 55%, 60%, and 65% groups. Rx: Prescription; IMRT: Intensity-modulated radiotherapy; RMS: Root mean square; CT: Computed tomography; Max: Maximum; Min: Minimum; GTV: Gross tumor volume.

Item	Setting details						
Prescription	Rx Dose (Gy): 43.000; Number of Fractions: 5						
IMRT constraints (Pareto)	Structure	Cost function	Parameter settings				
	GTV	Target Penalty	Prescription (Gy): 43.000; Minimum Volume (%): 95.00-99.98*				
		Quadratic Overdose**	Group	None	55%	60%	65%
			Maximum Dose (Gy)	-	78.182 Gy	71.667 Gy	66.154 Gy
			RMS Dose Excess (Gy): 0.020; Multicriterial +; Shrink Structures (cm): GTV 0.20				
	Patient (Body contour)	Conformality	Relative isoconstraint: 0.01; Margin Around Target: 8 cm; Multicriterial +				
		Quadratic Overdose	Maximum Dose (Gy): 43.000; RMS Dose Excess (Gy): 0.020; Multicriterial +; Shrink Structures (cm): GTV 0.20				
IMRT Prescription Parameters	Minimum CT Number: -200; Auto Flash Margin (cm): 0.20; Surface Margin (cm): 0.30; Beamlet Width (cm): 0.30; Target Margin: Normal (8 mm); Avoidance Margin: Normal (8 mm)						
Sequencing Parameters	Segment Shape Optimization: +; High Precision Leaf Positions: 20; Max Number of Arcs: 1; Max. # of Control Points per Arc: 1024; Min. Segment Width (cm): 0.50; Fluence Smoothing: Medium						
Calculation Properties	Grid Spacing (cm): 0.10; Dose Deposition Calculation: Medium; Statistical Uncertainty (%): 1.00 per calculation						

A unified prescription dose of 43.000 Gy in 5 fractions was assigned to each GTV margin. Each GTV coverage by the prescription dose was finally rescaled according to the minimum dose to the GTV minus 0.01 cc (*D*_V-0.01 cc_), and the rescaling ratio was recorded [13,28]. The total calculation time (tCT) was recorded from the optimization console on Monaco®, as described previously [13].

Treatment plans were evaluated using the plan with no internal dose constraint (None) as a baseline and compared with those using dose constraints (55%, 60%, and 65%). Plan comparisons were made based on evaluation criteria, including unique ones established in previous studies, as detailed in Table 3, considering the clinical issues of common evaluation metrics [13,25,28,29].

**Table 3:** Plan evaluation metrics used to compare dose distributions. *This index can also be interpreted as follows: the higher the value, the steeper the dose increase inside the GTV boundary, although high values may occur due to over-coverage of the GTV by a prescription isodose. GTV: Gross tumor volume; *D*_V-0.01 cc_: Minimum dose to a target volume (TV) minus 0.01 cc (D>95% for TV >0.20 cc; D95% for TV ≤0.20 cc); IDS: Isodose surface; *D*_0.01 cc_: Minimum dose to 0.01 cc receiving a near-maximum dose within the TV (D<5% for TV >0.20 cc; D5% for TV ≤0.20 cc); PIV: Prescription isodose volume; IIDV: Irradiated isodose volume; DeIIV: Minimum dose to the IIDV equivalent to a TV.

Evaluation Items	Metrics	Definitions	Interpretation
GTV dose inhomogeneity	GTV DV-0.01 cc % IDS (%)	GTV DV-0.01 cc (%) relative to the D0.01 cc (100%)	The lower the value, the more heterogeneous
GTV dose conformity	PIV spillage (cc)	IIDV of GTV DV-0.01 cc minus the GTV	The smaller the value, the better the conformity
	GTV DeIIV (%)	Minimum dose (%) to IIDV equivalent to GTV, relative to the GTV DV-0.01 cc (100%)	The closer to 100%, the better the conformity *
	GTV DeIIV coverage (%)	GTV coverage (%) by the DeIIV	The higher the value, the better the conformity
Steepness of dose decrease outside the GTV boundary	GTV + 2 mm DeIIV (%)	Minimum dose (%) to IIDV equivalent to GTV + 2 mm, relative to the GTV DV-0.01 cc (100%)	The lower the value, the steeper the dose decrease (gradient) outside the GTV boundary
Concentric lamellarity of dose gradient outside the GTV boundary	GTV + 2 mm DeIIV coverage (%)	GTV + 2 mm coverage (%) by the DeIIV	The higher the value, the better the concentric layering of the dose gradient outside the GTV boundary
Steepness of dose gradient outside the GTV	75% or 50% PIV spillage (cc)	IIDV of 75% or 50% of GTV DV-0.01 cc minus the GTV	The smaller the value, the steeper the dose gradient outside the GTV boundary
Steepness of dose increase inside the GTV boundary	GTV - 2 or 4 mm DeIIV (%)	Minimum dose (%) to IIDV equivalent to GTV - X mm, relative to the GTV DV-0.01 cc (100%)	The higher the value, the steeper the dose increase inside the GTV boundary
Concentric lamellarity of dose gradient inside the GTV boundary	GTV - 2 or 4 mm DeIIV coverage (%)	GTV - 2 or 4 mm coverage (%) by the DeIIV	The higher the value, the better the concentric layering of the dose gradient inside the GTV boundary

The GTV - 2 mm and GTV - 4 mm structures were created only for GTVs of ≥0.50 cc (34 lesions) and ≥1.51 cc (29 lesions), respectively, to ensure minimum meaningful volumes for evaluation [29].

Statistical analyses were performed using paired nonparametric tests, based on the results of the Shapiro-Wilk normality test. Box-and-whisker plots (BWPs) were used to show the distributions of each variable, where whiskers indicate the nearest values ≤1.5 times the IQR, and cross marks beyond the whiskers denote outliers >1.5 times the IQR. The Wilcoxon signed-rank test (WSRT) was adopted to compare two numerical variables with and without internal dose constraints. In addition, the Jonckheere-Terpstra (JT) test was used as a multigroup test to assess trends of increase or decrease among the four numerical variables with gradually increasing internal dose constraints. Statistical significance was considered at p<0.05 and expressed on a three-level scale: p<0.05 (*), p<0.01 (), and p<0.001 (*). Significant p-values were marked in blue in the figures. All analyses were performed using BellCurve for Excel® (Version 4.05; Social Survey Research Information Co., Ltd., Tokyo, Japan).

## Results

**Figure 1:**
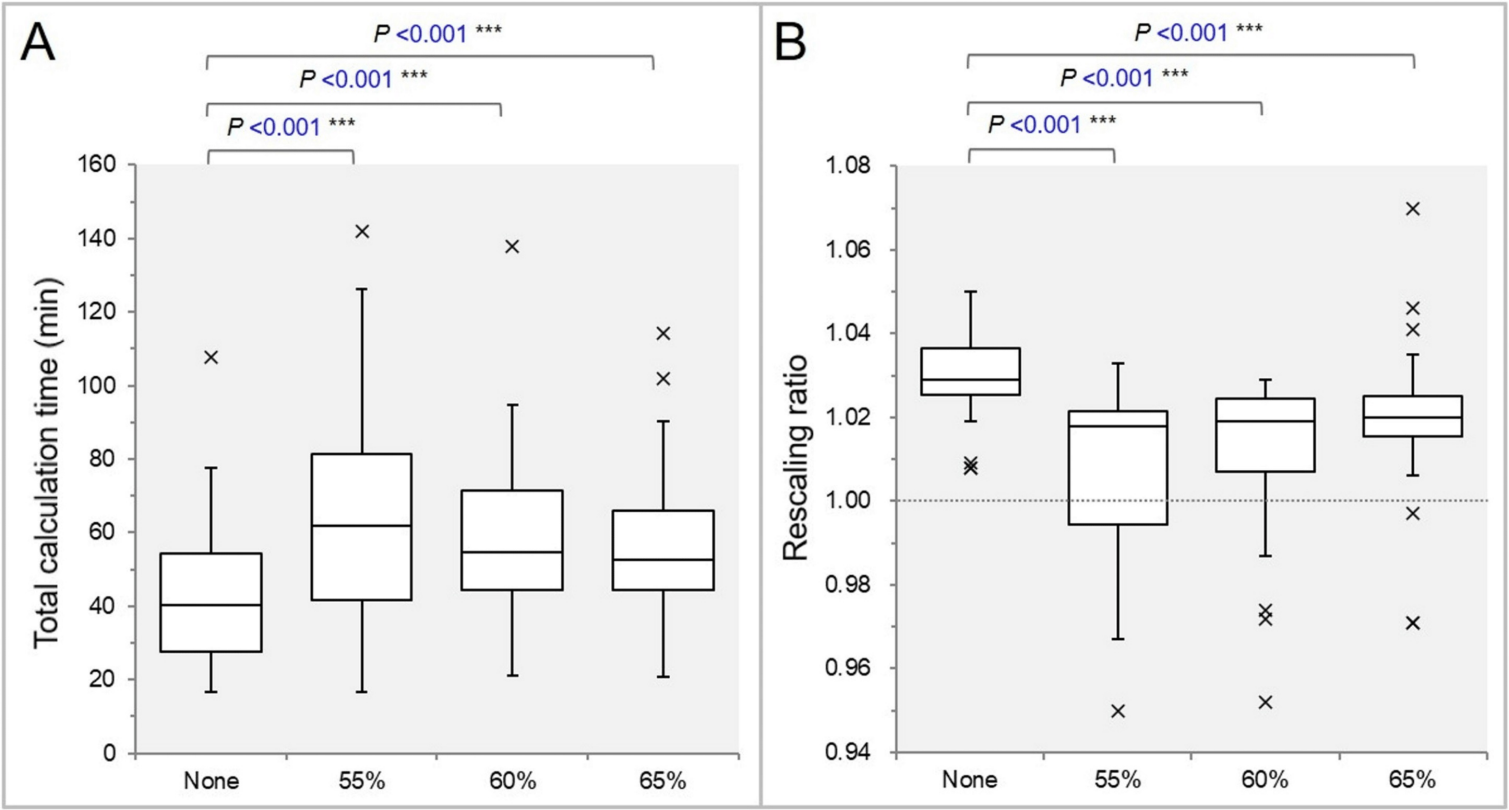
Comparisons of total calculation time and rescaling ratio. Box-and-whisker plots (BWPs) in panels (A) and (B) illustrate comparisons of total calculation time (A) and the rescaling ratio used to align the prescription dose after optimization (B) across the four groups. Results of the Wilcoxon signed-rank test (WSRT) are included. The dotted line in (B) indicates a value of 1.000, representing no need for rescaling.

The tCT was significantly shorter in the None group than in the 55%, 60%, and 65% groups (Figure 1A).

In particular, the tCT tended to be the longest in the 55% group (Figure 1A). The rescaling ratio was significantly higher in the None group, while the variance of the values was the smallest (Figure 1B).

**Figure 2:**
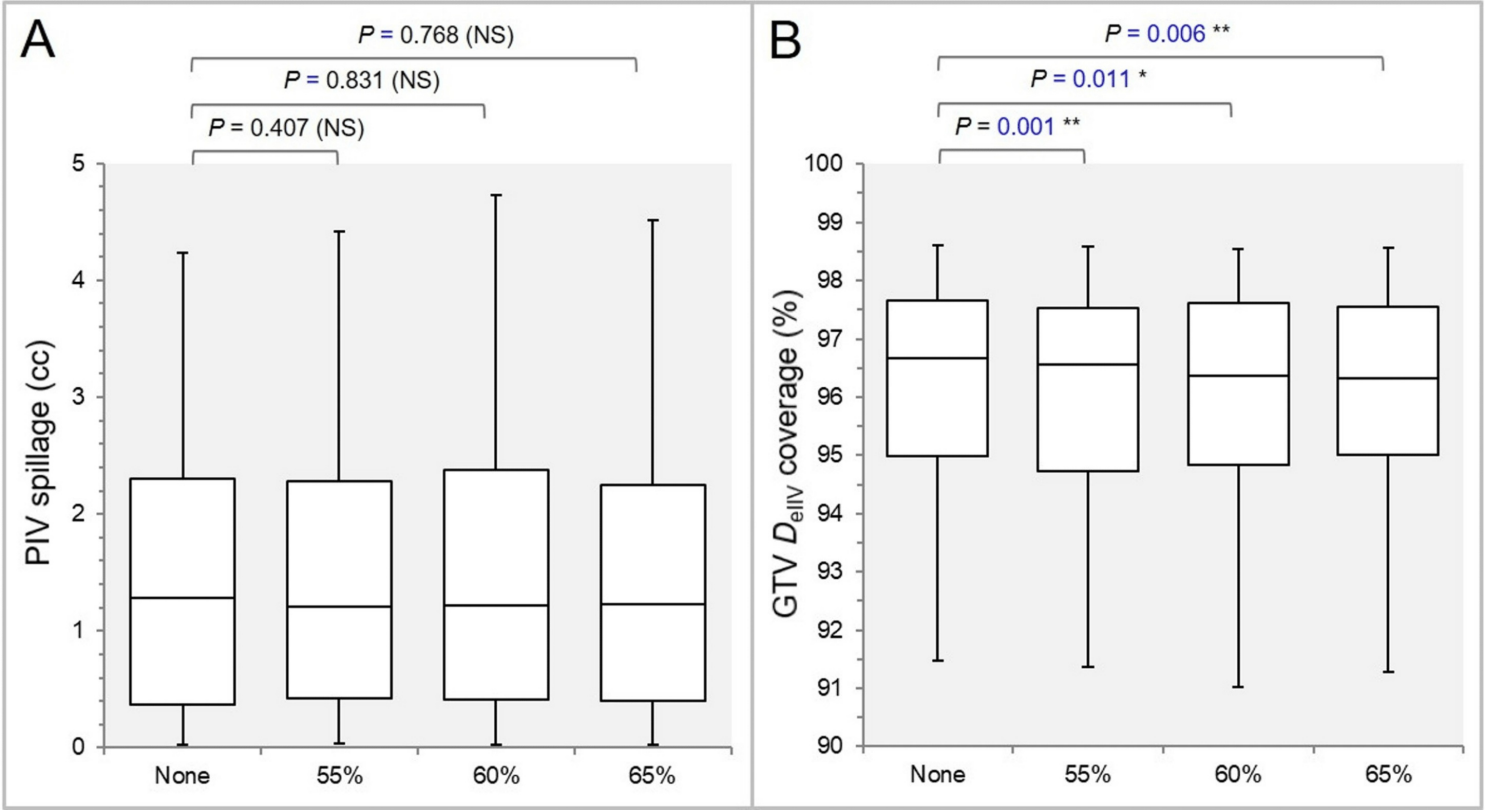
Comparisons of the GTV dose conformity. he images show BWPs (A, B) comparing PIV spillage volumes (A) and GTV coverage values by the *D*_eIIV_ (B), along with the results of the WSRT. GTV: Gross tumor volume; PIV: Prescription isodose volume; *D*_eIIV_: Minimum dose to the irradiated isodose volume (IIDV) equivalent to a target volume (TV); NS: not significant; BWPs: box-and-whisker plots; WSRT: Wilcoxon signed-rank test.

There were no significant differences in the prescription isodose volume (PIV) spillage (Figure 2A).

The volume in the None group was larger compared with the 55% and 60% groups in 54.3% of cases, while it was smaller in 57.1% of cases in the None group compared to the 65% group (Figure 2A). The GTV coverage value by the *D*_eIIV_, the minimum dose to the irradiated isodose volume (IIDV) equivalent to a GTV, was significantly higher in the None group (Figure 2B).

**Figure 3:**
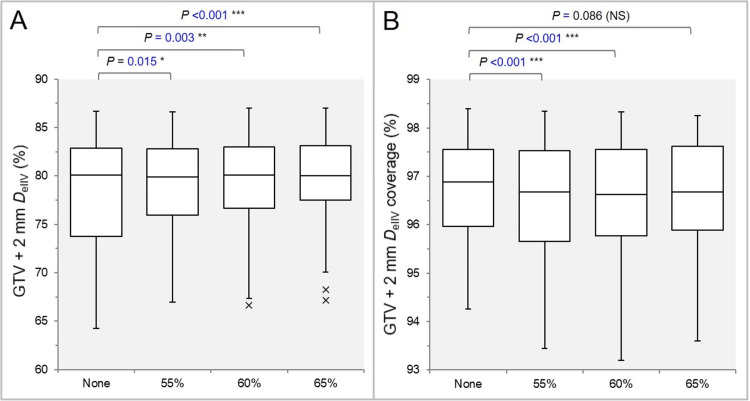
Comparisons of the appropriateness of dose attenuation margin outside the GTV boundary. The images show BWPs (A, B) comparing the GTV + 2 mm *D*_eIIV_ (%) relative to the GTV *D*_V-0.01 cc_ (100%) (A) and the coverage value of GTV + 2 mm by the *D*_eIIV_ (B), along with the results of the WSRT. GTV: Gross tumor volume; GTV + 2 mm: GTV evenly expanded by 2 mm; *D*_eIIV_: Minimum dose to the irradiated isodose volume (IIDV) equivalent to a target volume (TV); NS: Not significant; BWPs: Box-and-whisker plots; *D*_V-0.01 cc_: Minimum dose to a TV minus 0.01 cc; WSRT: Wilcoxon signed-rank test.

The *D*_eIIV_ of the GTV + 2 mm was significantly lower in the None group (Figure 3A), and it was <69.8% (too steep) in three, two, three, and two cases (GTV 0.33-0.72 cc) in the None, 55%, 60%, and 65% groups, respectively (Figure 3A) [25].

The GTV + 2 mm coverage value by the *D*_eIIV_ was significantly higher in the None group than in the 55% and 60% groups, and it was higher in 65.7% of cases in the None group compared to the 65% group (Figure 3B).

**Figure 4:**
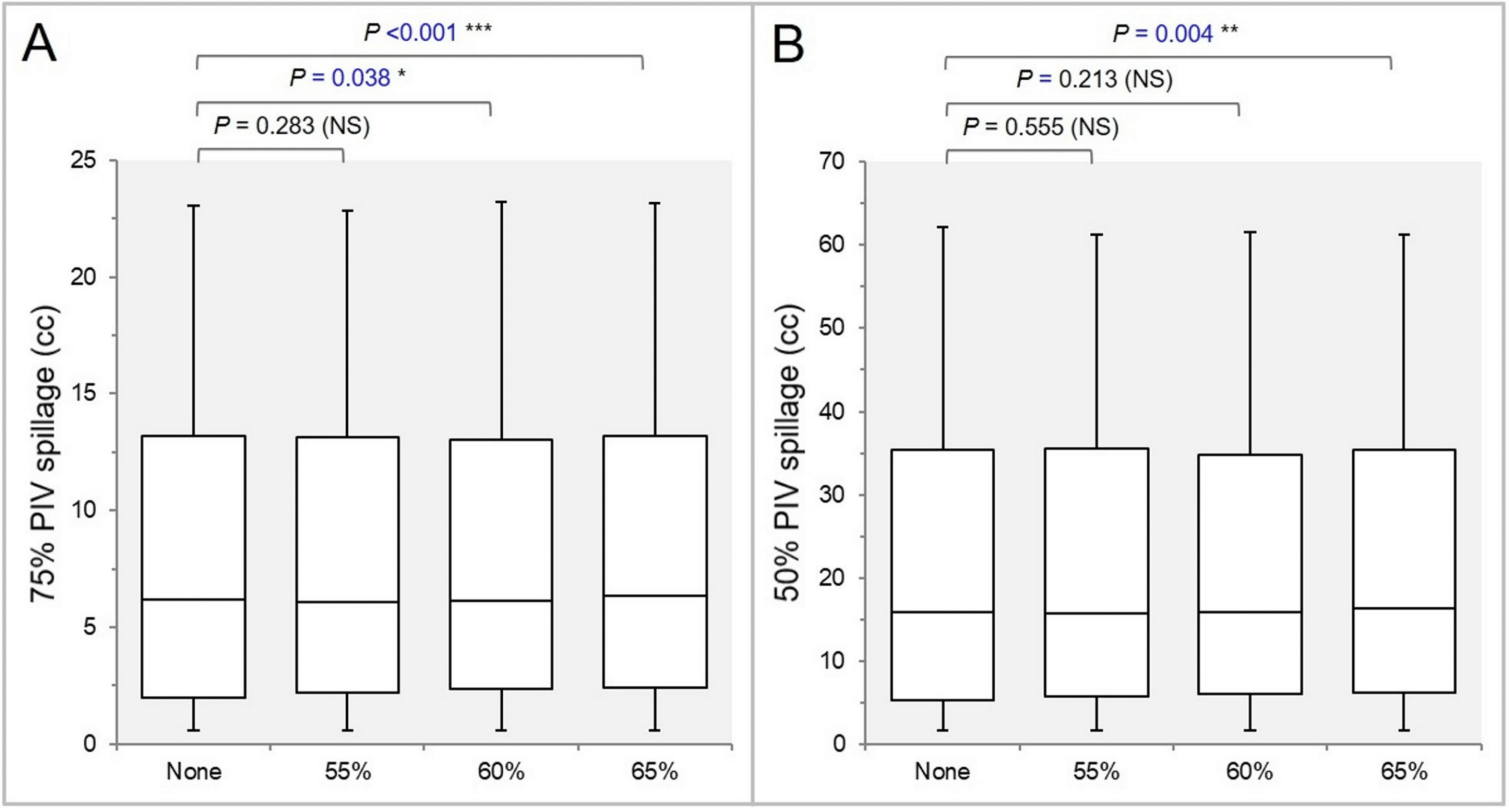
Comparisons of the steepness of the dose gradient outside the GTV boundary. The images show BWPs (A, B) comparing the 75% (A) and 50% (B) PIV spillage volumes, along with the results of the WSRT. GTV: Gross tumor volume; PIV: Prescription isodose volume; NS: Not significant; BWPs: Box-and-whisker plots; WSRT: Wilcoxon signed-rank test.

The 75% PIV spillage volume was significantly smaller in the None group than in the 60% and 65% groups, and it was smaller in 60.0% of cases in the None group compared to the 55% group (Figure 4A).

The 50% PIV spillage volume was significantly smaller in the None group than in the 65% group, and it was smaller in 54.3% and 62.9% of cases in the None group compared to the 55% and 60% groups, respectively (Figure 4B).

**Figure 5:**
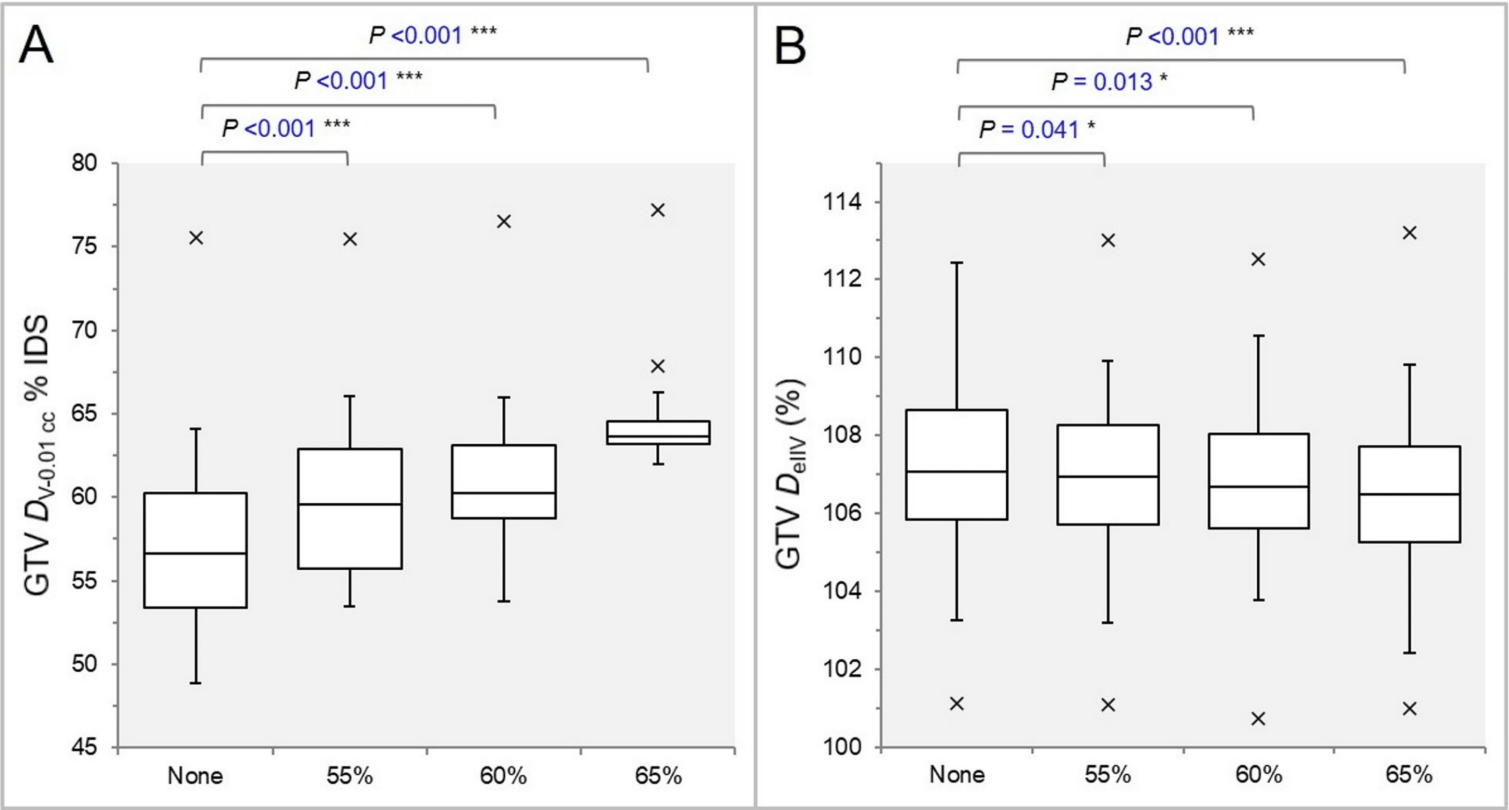
Comparisons of the GTV dose inhomogeneity and the steepness of dose increase just inside the prescription isodose surface. The images show BWPs (A,B) comparing the GTV *D*_V-0.01 cc_ (%) relative to the *D*_0.01 cc_ (100%) (A) and the GTV *D*_eIIV_ (%) relative to the *D*_V-0.01 cc_ (100%) (B), along with the results of the WSRT. GTV: Gross tumor volume; *D*_V-0.01 cc_: Minimum dose to a target volume (TV) minus 0.01 cc; IDS: Isodose surface; *D*_eIIV_: Minimum dose to the irradiated isodose volume (IIDV) equivalent to a TV; BWPs: Box-and-whisker plots; *D*_0.01 cc_: Minimum dose to 0.01 cc, receiving a near maximum dose, of a TV; WSRT: Wilcoxon signed-rank test.

The GTV dose was most inhomogeneous in the None group (Figure 5A), and the GTV *D*_eIIV_ was significantly higher in the None group (Figure 5B).

**Figure 6:**
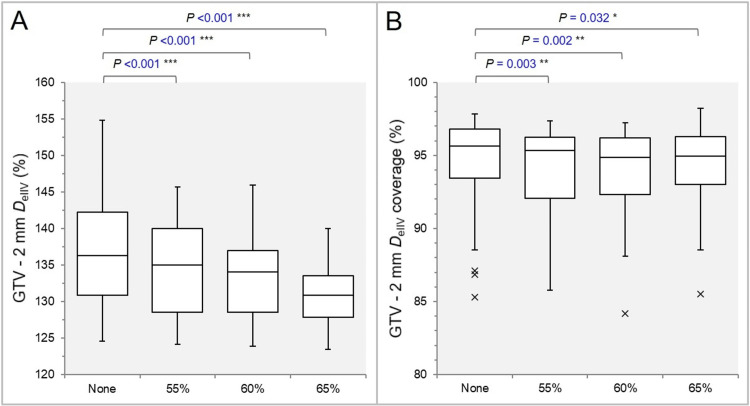
Comparisons of the steepness of dose increase and the concentric lamellarity at 2 mm inside the GTV boundary. The images show BWPs (A,B), along with the results of the JT test, for comparisons of the GTV - 2 mm *D*_eIIV_ (%) relative to the GTV *D*_V-0.01 cc_ (100%) (A) and the coverage value of GTV - 2 mm by the *D*_eIIV_ (B), along with the results of the JT test and WSRT. GTV: Gross tumor volume; GTV -2 mm: GTV evenly reduced by 2 mm; *D*_eIIV_: Minimum dose to the irradiated isodose volume (IIDV) equivalent to a target volume (TV); NS: Not significant; BWPs: Box-and-whisker plots; *D*_V-0.01 cc_: Minimum dose to a TV minus 0.01 cc; WSRT: Wilcoxon signed-rank test.

The GTV -2 mm *D*_eIIV_ was significantly higher in the None group (Figure 6A), and the coverage value by the *D*_eIIV_ was significantly higher in the None group (Figure 6B).

**Figure 7:**
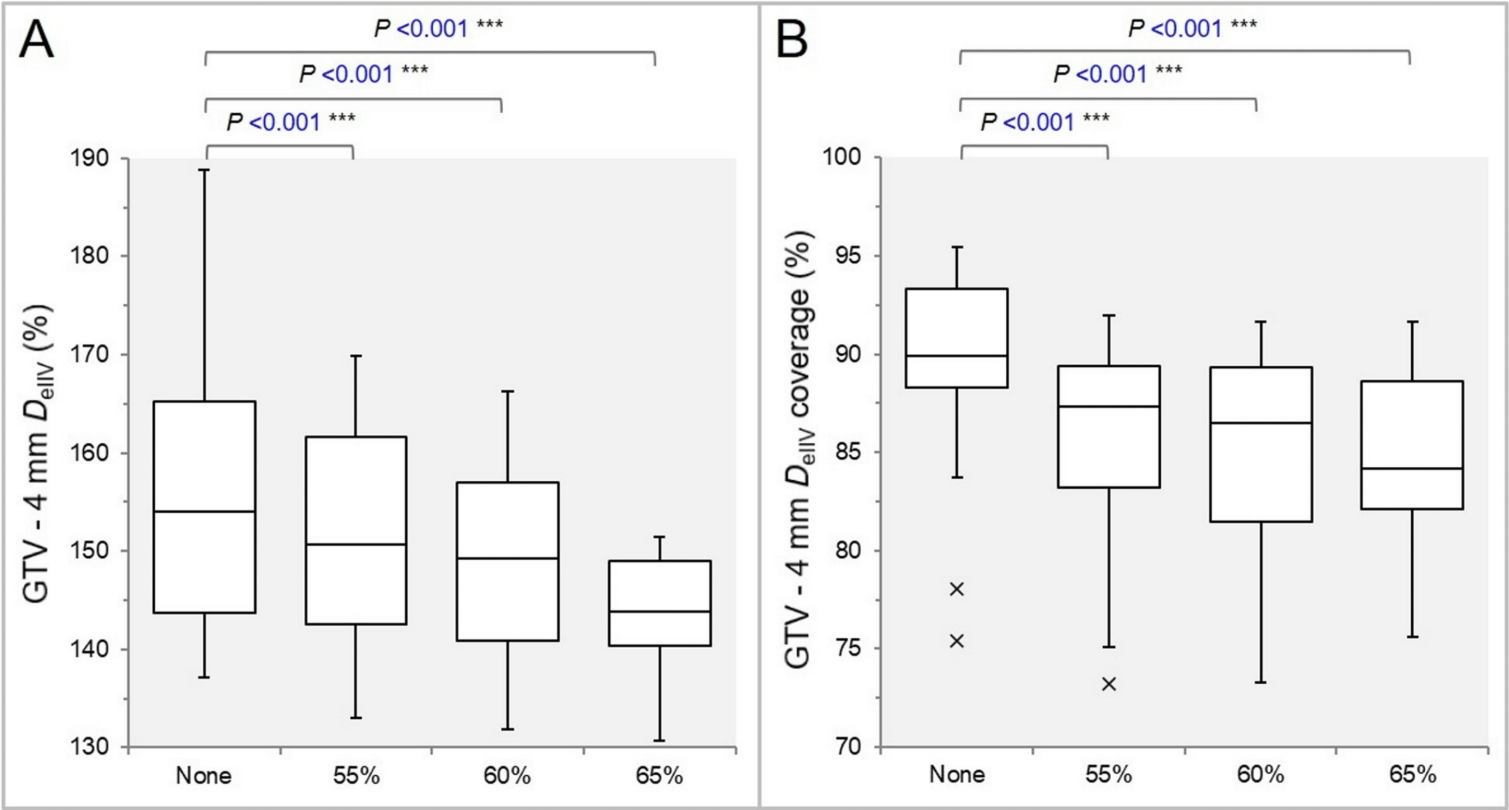
Comparisons of the steepness of dose increase and the concentric lamellarity at 4 mm inside the GTV boundary. The images show BWPs (A,B) for comparisons of the GTV - 4 mm *D*_eIIV_ (%) relative to the GTV *D*_V-0.01 cc_ (100%) (A) and the coverage value of GTV - 4 mm by the *D*_eIIV_ (B), along with the results of the WSRT. GTV: Gross tumor volume; GTV - 4 mm: GTV evenly reduced by 4 mm; *D*_eIIV_: minimum dose to the irradiated isodose volume (IIDV) equivalent to a target volume (TV); NS: Not significant; BWPs: Box-and-whisker plots; *D*_V-0.01 cc_: Minimum dose to a TV minus 0.01 cc; WSRT: Wilcoxon signed-rank test.

The GTV -4 mm *D*_eIIV_ was significantly higher in the None group (Figure 7A), and the coverage value by the *D*_eIIV_ was significantly higher in the None group (Figure 7B).

The increasing/decreasing trends in the plan evaluation metrics with increasing maximum dose constraints are shown in Table 4.

**Table 4:** Increase/decrease trends in plan evaluation metrics with increasing maximum dose constraints. The results are based on the Jonckheere-Terpstra (JT) test. PIV: Prescription isodose volume; GTV: Gross tumor volume; *D*_eIIV_: Minimum dose to the irradiated isodose volume (IIDV) equivalent to a target volume (TV); *D*_V-0.01 cc:_ Minimum dose to a TV minus 0.01 cc (*D*_>95%_ for TV >0.20 cc, *D*_95%_ for TV ≤0.20 cc); IDS: Isodose surface; NS: Not significant.

Metrics	Trend	P-value
Total calculation time	Increase	p = 0.008 **
Rescaling ratio	Decrease	p < 0.001 ***
PIV spillage	Decrease	p = 0.500 (NS)
GTV D_eIIV_ coverage (%)	Decrease	p = 0.399 (NS)
GTV + 2 mm D_eIIV_	Increase	p = 0.176 (NS)
GTV + 2 mm D_eIIV_ coverage	Decrease	p = 0.429 (NS)
75% PIV spillage	Increase	p = 0.356 (NS)
50% PIV spillage	Increase	p = 0.400 (NS)
GTV D_V-0.01_ cc % IDS (%)	Increase	p < 0.001 ***
GTV D_eIIV_	Decrease	p = 0.076 (NS)
GTV - 2 mm D_eIIV_	Decrease	p < 0.001 ***
GTV - 2 mm D_eIIV_ coverage	Decrease	p = 0.224 (NS)
GTV - 4 mm D_eIIV_	Decrease	p < 0.001 ***
GTV - 4 mm D_eIIV_ coverage	Decrease	p < 0.001 ***

**Figure 8:**
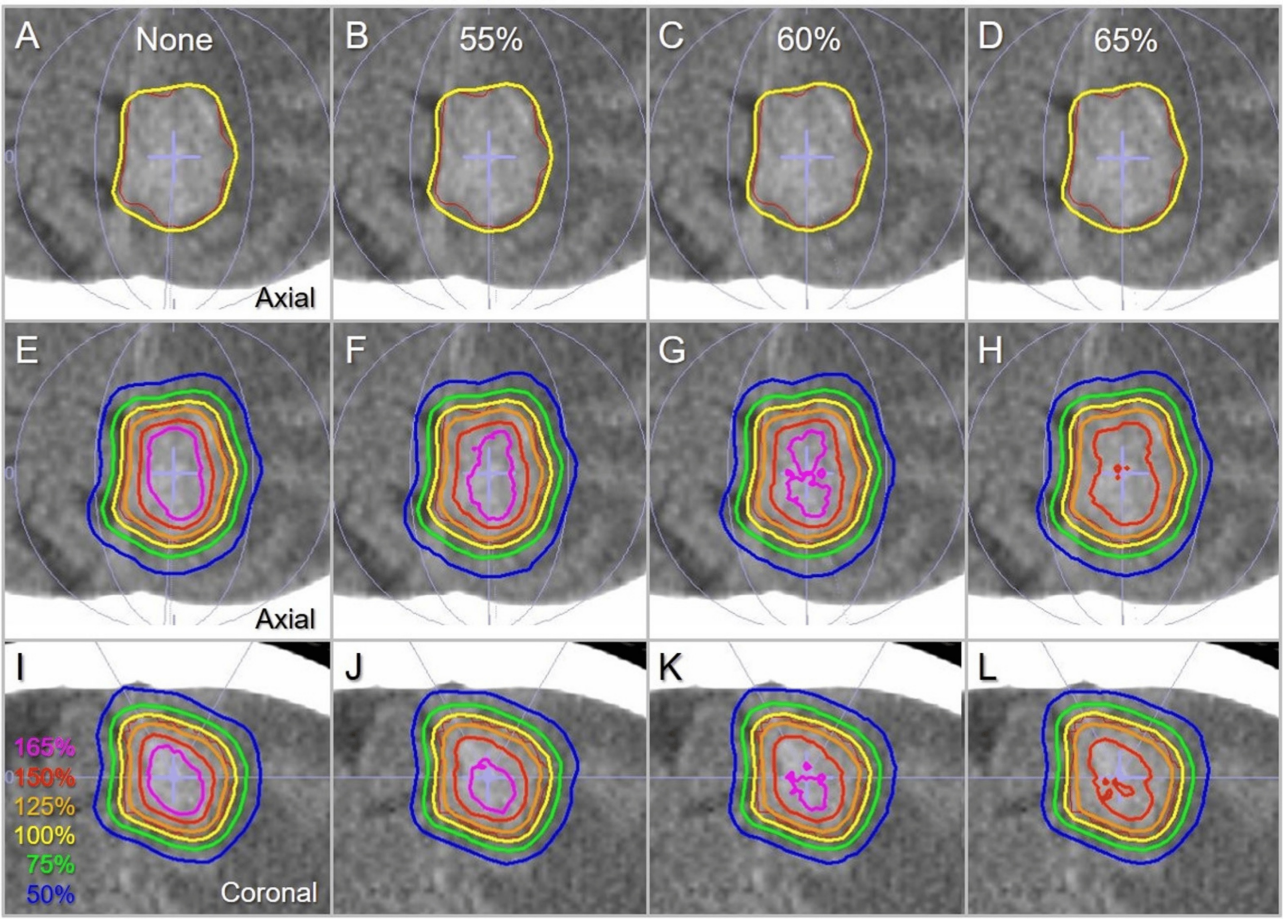
Comparison of the dose distributions for a GTV of 7.14 cc. The images show computed tomographic scans of a patient with a brain metastasis (BM) located in the left frontoparietal lobes (A-F), with superimposed representations of the GTV outline (red), arc arrangements (light purple), and representative isodose lines from the None, 55%, 60%, and 65% plans. Axial views (A-H) and coronal views (I-L) are displayed. The isodose lines are presented as relative values, using the GTV D_V-0.01_ cc (D99.86%) as 100% (yellow). After rescaling the GTV coverage, the isodose values of the GTV D_V-0.01 cc_ relative to the D_0.01 cc_ (100%) are 55.4%, 57.7%, 58.4%, and 62.0% in the None, 55%, 60%, and 65% plans, respectively. GTV: Gross tumor volume; BM: Brain metastasis; D_V-0.01 cc_: Minimum dose to a target volume (TV) minus 0.01 cc; D_0.01 cc_: Minimum dose to 0.01 cc of a TV, receiving a near-maximum dose.

Figure 8 shows a comparison of the representative isodose distributions for the GTV of 7.14 cc, which was previously described in a case report [30].

The differences in the 150% and 165% isodose lines are clearly visible with increasing internal dose constraints.

## Discussion

The maximum dose constraints in this study were assigned to substantially high doses of >1.5 times the prescription dose, which was sufficient to allow for ≥66.7% IDS coverage (e.g., 70%). However, all three dose constraints led to significant increases in optimization time. Despite the constraints on the high-dose area near the GTV center, the steepness of the dose increase 2-4 mm inside the GTV boundary decreased significantly with increasing maximum dose constraints, which may impair early and sufficient tumor shrinkage after the start of SRS, especially in large lesions [15,16]. In addition, the dose constraints vitiated the concentric lamellarity of dose gradients from the GTV boundary to its interior and exterior [25,29]. In all four groups, the dose attenuation margin was too steep in some small lesions [25].

In general, the steepness of the dose fall-off outside the target boundary competes with target dose homogeneity [15,24]. Prioritizing target dose uniformity requires larger leaf margins to the target boundary and compromises dose conformity and gradients. The Monaco® system actively tolerates high doses within the target to maximize dose conformity and the steepness of dose fall-off outside the target [24]. Segment shape optimization develops non-conformal beam segments that avoid extending beyond the GTV border as much as possible. Many segments tend to overlap within the GTV, leading to the formation of internal high-dose areas. However, maximum dose constraints are likely to conflict with the optimization process. Stronger dose constraints are more likely to create larger segments that extend beyond the GTV boundary, compromising dose conformity and gradients.

The reproducibility of the maximum dose, i.e., D_0.001 cc_, of a GTV is low due to inter- and intra-fractional setup errors and/or cranial motions, especially with frameless multi-fraction SRS [13]. The D_≥0.01 cc_, equivalent to 10 voxels or more, is more likely to affect treatment outcomes than D_0.001 cc_ [13]. The doses at 2-4 mm inside the GTV boundary may have a greater impact on early tumor response than the near-maximum dose such as D_0.01 cc_ [16,29]. In practice, there may be little clinical significance in keeping the GTV or PTV dose inhomogeneity strictly constant (e.g., 70% IDS coverage) for VMA planning. Individual GTV dose inhomogeneities are determined as a result of simple optimization that prioritizes dose conformity and the steepness of dose fall-off outside the GTV [13,24]. Optimal target dose heterogeneity varies with lesion size and shape.

In general SRS practice, such as dose prescription to the 70% IDS encompassing a PTV with a ≥1 mm margin, GTV dose inhomogeneity is inevitably reduced with <70% IDS coverage, and the GTV marginal dose, e.g., D_V-0.01 cc_, also fluctuates substantially [28]. In addition, the dose fall-off outside the GTV boundary tends to be less steep than optimization based on the GTV boundary. In contrast, by prescribing the BED at the GTV boundary at a constant level with VMA optimization targeting the GTV boundary, an appropriate dose attenuation margin is achieved, with the BED at ≥2 mm outside the GTV boundary being higher for larger lesions [25]. Thus, VMA optimization based on the GTV boundary without any internal dose constraints is optimal and recommended, especially for large lesions. It is necessary to consider methods other than internal dose constraints to adjust overly steep dose attenuation margins in small GTVs [25].

Study limitations

In this study, even though optimization aimed to achieve ≥60% IDS coverage, normalization actually resulted in <60% IDS coverage in nearly half of the cases. Similar results were observed in the 55% and 65% groups. The application of stricter dose constraints aiming for ≥70% IDS coverage, i.e., ≤142.86% of the prescription dose, was also considered; however, this was expected to further impair dose conformity and the concentric lamellarity of dose gradients from the GTV boundary to both the exterior and interior. Although the VMA optimizations were performed under uniform settings, different results may be obtained by adjusting the weights between the cost functions. Excessively high internal GTV doses, approximately twice as high as the marginal dose, may lead to tumor swelling and/or intratumoral bleeding. Significant tumor shrinkage during ≥5-fraction SRS with an internal steep dose increase would inevitably lead to a substantial increase in the surrounding brain dose [24]. In actual clinical practice, the effectiveness and safety of SRS with an extremely inhomogeneous GTV dose must be carefully monitored [24]. This study was limited to analysis at the treatment planning stage of SRS, and the clinical usefulness of optimization without internal dose constraints within the GTV needs to be demonstrated in actual clinical cases.

## Conclusions

In VMA-based SRS planning, maximum dose constraints within the GTV, exceeding 1.5-1.8 times the prescription dose, impaired GTV dose conformity, the appropriateness of the dose attenuation margin, and the steepness and concentric lamellarity of dose gradients both outside and inside the GTV boundary. Furthermore, these constraints hindered the efficiency of VMA optimization in maximizing dose conformity and gradient sharpness. Dose constraints intended to ensure ≥55-65% IDS coverage are generally not recommended. Alternative methods should be considered to adjust for excessively steep dose attenuation margins in small GTVs.
